# Challenges for malaria elimination in Brazil

**DOI:** 10.1186/s12936-016-1335-1

**Published:** 2016-05-20

**Authors:** Marcelo U. Ferreira, Marcia C. Castro

**Affiliations:** Department of Parasitology, Institute of Biomedical Sciences, University of São Paulo, Av. Prof. Lineu Prestes 1374, Cidade Universitária, São Paulo, SP 05508-900 Brazil; Department of Global Health and Population, Harvard T.H. Chan School of Public Health, 665 Huntington Avenue, Building I, Room 1113, Boston, MA 02115 USA

**Keywords:** Malaria, Malaria control, Malaria elimination, Brazil, Malaria control challenges

## Abstract

Brazil currently contributes 42 % of all malaria cases reported in the Latin America and the Caribbean, a region where major progress towards malaria elimination has been achieved in recent years. In 2014, malaria burden in Brazil (143,910 microscopically confirmed cases and 41 malaria-related deaths) has reached its lowest levels in 35 years, *Plasmodium falciparum* is highly focal, and the geographic boundary of transmission has considerably shrunk. Transmission in Brazil remains entrenched in the Amazon Basin, which accounts for 99.5 % of the country’s malaria burden. This paper reviews major lessons learned from past and current malaria control policies in Brazil. A comprehensive discussion of the scientific and logistic challenges that may impact malaria elimination efforts in the country is presented in light of the launching of the Plan for Elimination of Malaria in Brazil in November 2015. Challenges for malaria elimination addressed include the high prevalence of symptomless and submicroscopic infections, emerging anti-malarial drug resistance in *P. falciparum* and *Plasmodium vivax* and the lack of safe anti-relapse drugs, the largely neglected burden of malaria in pregnancy, the need for better vector control strategies where *Anopheles* mosquitoes present a highly variable biting behaviour, human movement, the need for effective surveillance and tools to identify foci of infection in areas with low transmission, and the effects of environmental changes and climatic variability in transmission. Control actions launched in Brazil and results to come are likely to influence control programs in other countries in the Americas.

## Background

Malaria is endemic to 21 countries in the region of the Americas, with 389,390 laboratory-confirmed cases and 87 malaria-related deaths reported to the Pan American Health Organization (PAHO) in 2014. Nearly 20 % of the local population is exposed to some risk of infection [1]. The region comprises eight of the 34 endemic countries worldwide with a national policy for malaria elimination—namely, Argentina, Costa Rica, El Salvador, Dominican Republic, Mexico, Nicaragua, Panama, and Paraguay [2]; moreover, eighteen of the region’s 21 malaria-endemic countries have expressed commitment towards malaria elimination. Fourteen malaria-endemic countries in the region are on track to achieve a 75 % reduction in their case incidence rates between 2000 and 2015, as called for by the United Nations’ Millennium Development Goals target 6.C [1].

Brazil recorded a 76.8 % decrease in malaria incidence between 2000 and 2014, and now contributes 42 % of all malaria cases reported in the Americas [1]. Transmission remains entrenched in the Amazon Basin, which accounts for 99.5 % of the country’s malaria burden. Here, past and current malaria control policies and achievements in Brazil are reviewed, and key scientific and logistic challenges for eliminating residual malaria in the Amazon are discussed.

### A brief history of malaria control in Brazil

Malaria control in Brazil started effectively at the beginning of the twentieth century [3]. The first anti-malaria campaign was implemented in 1905, during the construction of the port of Santos in the southeastern State of São Paulo, by the well-known physician Carlos Chagas (who few years later described Chagas’ disease). An association between housing conditions and the transmission of the disease was soon observed. Mosquito collections inside the houses in the construction area were done every 8 days, screens installed in all windows, and in-house fumigation with sulphur adopted [4–6]. After a month, the daily number of malaria episodes decreased dramatically, with no new cases being observed after 3 months [4]. It was the first time that a measure aimed at killing the adult mosquito was adopted for malaria control [7]. Its success originated the concept that malaria is a ‘household infection’ (a disease acquired mostly inside the houses), accepted worldwide a few decades later. This concept became the basis for interventions such as indoor residual spraying (IRS) and the use of bed nets.

Between 1923 and 1925, a team led by Mark Boyd analysed four malarious areas in Rio de Janeiro State in order to propose alternatives for malaria control according to their environmental characteristics and the available financial resources [3]. Engineering projects to promote environmental management, such as the construction of drainage systems and filling marshes, led to major reductions in malaria transmission in coastal areas of southeast Brazil in the 1920s and 1930s [8, 9].

In 1930, an African malaria vector (originally identified as *Anopheles gambiae* but later shown to be *Anopheles arabiensis* [10]) was discovered in the Northeast of Brazil, most likely brought by a ship coming from Senegal, progressively spreading along the coast. In only 8 months in 1938–1939 there were 150,000 cases of malaria, and 14,000 deaths [11]. With the support from the Rockefeller Foundation, a massive control operation was implemented at the end of 1938. Approximately 4000 workers were employed during 19 months of control. The costs of the operation reached US$1.8 million, 87 % financed by the Brazilian government. Measures of control included monthly house spraying, early case detection and rapid treatment, spraying of cars and trucks leaving or entering the endemic area, elimination of breeding sites, ditching of subsurface water areas, and use of chemical larvicides. In 1940, *An. arabiensis* had been eliminated from Brazil [3, 12]. A similar campaign was repeated in Egypt in 1943–1944 [13]. These two successful campaigns inspired the eradication strategy that marked the World Health Organization (WHO) malaria agenda between 1955 and 1969 [14, 15].

### Large-scale malaria control and attempted elimination (1940–1970)

**Fig. 1:**
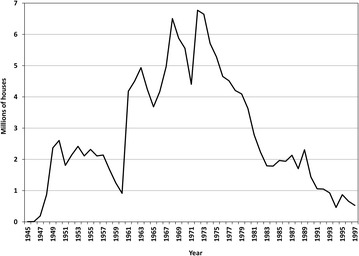
Annual number of houses sprayed with dichloro-diphenyl-trichloroethane (DDT) in Brazil between 1945 and 1997. Data for the years 1945–1959 and 1965–1986 were obtained from the Brazilian Institute of Geography and Statistics, available at: http://seculoxx.ibge.gov.br/pt/populacionais-sociais-politicas-e-culturais/busca-por-palavra-chave/saude/985-malaria. Data for the years 1960–1964 were extracted from PAHO/WHO reports [199]. Data for 1987–1997 were extracted from Loiola [52]

In the early 1940s, two-thirds of Brazil’s 40 million inhabitants lived in malaria-endemic areas. Six to eight million infections, and 80,000 malaria-related deaths, were estimated to occur each year [16, 17]. At that time, nationwide anti-malarial campaigns were initiated, following the establishment of the National Malaria Service [18]. Large-scale house spraying with dichloro-diphenyl-trichloroethane (DDT) started in 1947, after a few pilot studies in Amazonian cities since 1945 [18]. Over the next decade, DDT spraying was extended to two million houses (Fig. 1), with a dramatic effect on malaria transmission. In 1957, only 250,000 malaria cases were estimated to occur in a population of 62 million [17].

**Fig. 2:**
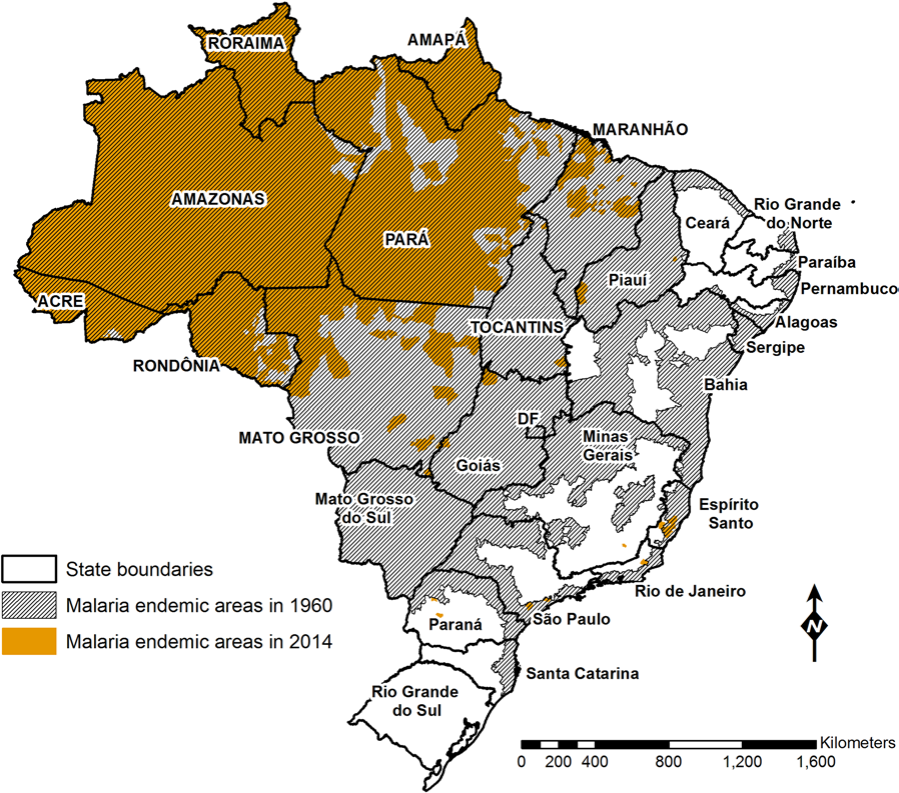
Extension of the malaria endemic areas in Brazil in 1960 and 2014. The *figure* shows how the malaria map in Brazil shrunk between 1960 and 2014. Currently, transmission is virtually limited to the Amazon Basin, an area that covers 60 % of the Brazilian territory and houses 13.4 % of the country’s population. States that compose the Amazon region have their names written in* uppercase*. Data obtained from the National Malaria Prevention and Control Program, Ministry of Health of Brazil

**Fig. 3:**
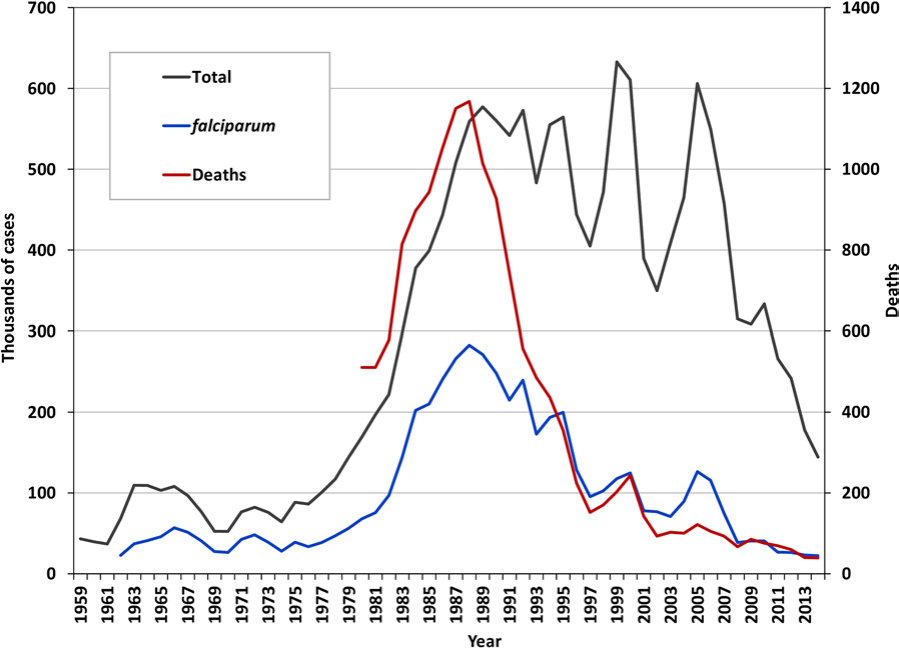
Annual number of laboratory-confirmed malaria cases reported in Brazil from 1959 to 2014. The total number of cases, those due to *Plasmodium falciparum*, and the number of malaria-related deaths are shown. Data obtained from the National Malaria Prevention and Control Programme, Ministry of Health of Brazil

Brazil’s malaria map shrank considerably between 1950 and 1970 (Fig. 2). Malaria was virtually eliminated in the Northeastern, Southeastern, and Southern regions of the country, as well as in most of the Center-West region. Malaria elimination efforts initiated in 1958 by the Workgroup for Malaria Control and Eradication were later scaled up by the Malaria Eradication Campaign, officially implemented in 1965 [17], adopting the Global Malaria Eradication Programme strategies of the WHO [19]. First, the DDT house-spraying programme, which had been declining since 1957, was strengthened to reach up to 6.5 million houses in the late 1960s (Fig. 1). During the attack phase, which involved the enactment of anti-larval, insecticidal, and anti-malarial measures for 3–5 years in order to interrupt malaria transmission, all houses in the endemic area should be periodically sprayed with DDT for 3–5 years. Moreover, routine active and passive case detection of febrile cases was implemented, with laboratory diagnosis followed by chloroquine (CQ) treatment and notification of 37,000–110,000 laboratory-confirmed infections each year over the 1960s (Fig. 3).

In the 1950s, Brazil pioneered another initiative for malaria control in hard-to-reach populations: the use of CQ added to cooking salt as a chemoprophylaxis strategy (Pinotti’s method) [20, 21]. Deployment of CQ-medicated or “chloroquinized” salt became a major component of the malaria elimination strategy in Brazil (between 1959 and 1961), as well in Guyana, Tanzania, Ghana, and Indonesia (between 1959 and 1965) [22, 23]. Major concerns regarding this strategy included its effectiveness, the risk of selecting CQ-resistant parasites, and changes in taste and colour of table salt depending on storage conditions [20, 23–25].

The smallest number of malaria cases ever recorded in the country (36,900) was registered in 1961 (Fig. 3). In 1970, 52,469 infections were diagnosed in Brazil, 60 % of them in the Amazon Basin [16]. With 59.7 % of the country’s territory, the Amazon had only 4 % of Brazil’s 90 million inhabitants enumerated by the 1970 population census. However, the demographic composition of the Amazon started to change at a fast pace, following efforts to integrate the area with the rest of the country, which brought about major increases in malaria transmission [26, 27].

### Resurgent malaria on the Amazon frontier (1970–1990)

Following the implementation of a military dictatorship in Brazil in 1964, political and military factors drove the ideals of national security and integration as part of larger geopolitical strategy [27, 28]. Road construction facilitated the implementation of mining, timber extraction, cattle ranching, and farming settlements in the Amazon. Waves of migrants, mostly from the malaria-free South, Southeast and Northeast regions (and thus malaria naïve), responded to federal government incentives: according to the 1980 census, the Amazonian states of Pará, Rondônia, Amazonas, Mato Grosso, Amapá, Acre, and Roraima received nearly one million immigrants during the 1970s [29].

Between 1970 and 1983, one-third of all farming settlements opened by the National Institute for Land Reform (INCRA) in the Amazon were located in the State of Rondônia [30]. While the whole population of the country experienced a yearly growth rate of 2.5 % during the 1970s, Rondônia registered an astonishing yearly growth rate of 15 %, with even higher increases in the rural area [31, 32]. Population growth continued during the 1980s after the Northwest Region Integrated Development Programme (POLONOROESTE), launched in 1981, partly financed by the World Bank [33]. In addition, gold prospecting and mining activities in southern Pará State attracted a flood of migrants, dramatically increasing the population of remote areas with no health infrastructure [29]. As a result, from 1980 to 1991, the country registered a population growth rate of 1.8 %, the states comprising the legal Amazon a rate of 3.25 %, and Rondônia’s population grew at 7.4 %.

The most important impacts of this massive human influx were significant environmental changes due to deforestation, and a dramatic increase in malaria incidence [29, 34]. With regards to malaria, a new concept of “frontier malaria” was proposed to characterize transmission in the Amazon [26, 35, 36]. Frontier malaria has a temporal pattern that can be summarized in three phases. First, the epidemic phase, marked by intense outbreaks observed for about 3 years after initial settlement. Outbreaks result from a combination of factors that include deforestation, which creates or expands breeding habitats of the main local malaria vector, *Anopheles darlingi* [37], close to colonists’ ramshackle houses in the fringes of the rainforest [38–40], lack of acquired immunity among most of the settlers, precarious habitat conditions that offered no protection against mosquitoes, and lack of adequate knowledge of the disease, among others. Second, the transition phase, characterized by gradual declines in malaria transmission observed over the following years, as the farming settlement becomes consolidated, with less environmental changes, better structured communities, improved housing, and better access to healthcare [35, 41, 42]. Finally, in the endemic phase, malaria transmission reaches lower and stable levels. In this setting, *P. falciparum* typically predominates in newly opened settlements, being progressively replaced by *P. vivax* in later stages [26].

In addition to these environmental and demographic changes, the late 1970s and the 1980s were one of the most difficult periods in Brazil’s recent economic history. Annual inflation rate was 110 % in 1980, jumping to 1783 % in 1989. All social indicators deteriorated during this period, including those related to basic health [43]. It was under this scenario that a tenfold increase in malaria incidence was observed between 1970 and 1985; in 1985 two states in the Amazon, Rondônia and Pará, accounted for 73 % of all malaria cases [29]. On average, more than half a million cases were observed annually during the 1990s, with a peak of 632.8 thousand cases in 1999.

### Changes in malaria species distribution

Following the concept of frontier malaria, Fig. 3 shows marked temporal changes in the relative contribution of *P. falciparum* to the malaria burden in Brazil. *Plasmodium falciparum* accounted for 12 % of all malaria infections diagnosed in Brazil in 1961, but this proportion increased over the following years. Between 1966 and 1973, and again between 1983 and 1989, similar proportions of slide-confirmed *P. falciparum* and *P. vivax* infections were diagnosed countrywide. Because the transmission of *P. vivax* species maintained an upward trend in the 1990s, while that of *P. falciparum* declined steadily, the proportion of falciparum malaria cases decreased since then. Of note is the fact that malaria-related deaths follow the same pattern as the contribution of falciparum malaria, and the decline in the proportion of falciparum cases was accompanied by a decline in the number of malaria-related deaths (Fig. 3). *Plasmodium vivax* now causes more than 84 % of all malaria infections diagnosed in Brazil. There is little documented transmission (less than 0.1 % of all cases) of *Plasmodium malariae* countrywide, although molecular techniques have revealed the presence of this species in 9–12 % of malaria patients in selected settings [44, 45].

The recent predominance of *P. vivax* in Brazil may be partially explained by biologic features that render this species less responsive than *P. falciparum* to control strategies based solely on early diagnosis and prompt treatment of blood-stage infections. First, low-density *P. vivax* infections are common, especially in areas approaching elimination, making laboratory diagnosis particularly difficult [46]. Second, parasites may persist in human hosts for several months as hypnozoites, the dormant liver stages that may eventually cause relapses [47].

Anti-malarial drugs that target both blood and liver stages are needed for the radical cure of vivax malaria, but primaquine (PQ), the only licensed anti-malarial with hypnozoitocidal activity, requires a relatively long treatment course (7–14 days) and can cause severe hemolysis in patients with glucose-6-phosphate dehydrogenase (G6PD) deficiency [48]. Furthermore, *P. vivax* transmission is greatly facilitated by the early production of infective stages, mature gametocytes. In fact, most vivax malaria patients have gametocytaemia detected by microscopy by the time they seek treatment [49], and virtually all *P. vivax* infections in Brazil, either symptomatic or asymptomatic, comprise gametocyte-specific *pvs25* gene transcripts detectable with sensitive molecular techniques [50].

### Malaria control on the Amazon frontier

In 1989, the Ministry of Health of Brazil launched the Amazon Basin Malaria Control Programme (PCMAM) to reduce resurgent malaria transmission and prevent its spread to non-endemic areas [51]. PCMAM was in operation until mid-1996, and its main strategy (earlier and more aggressive treatment of malaria cases to reduce transmission and mortality) had a clear short-term impact. Overall malaria incidence decreased by 60 % between 1989 and 1996, with the proportion of *P. falciparum* infections decreasing from 47 to 29 %. The network of malaria diagnosis and treatment outposts was greatly expanded across the Amazon to provide a CQ treatment to all suspected cases until diagnostic test results became available, followed by quinine or mefloquine for slide-confirmed *P. falciparum* infections [51]. House spraying with DDT, however, had been gradually phased out since the early 1980s [18, 29, 52], and DDT was officially banned from public health use in Brazil in 1998 [53]. Of note is the improvement in the information system that in 1993 gathered data from 98 % of the municipalities considered as having the potential for high malaria risk [51].

The gains associated with PCMAM were lost over the next few years, culminating with 637,000 microscopically confirmed malaria cases recorded in 1999 (Fig. 3). As a consequence, in 2000 the Ministry of Health implemented a comprehensive plan to reduce overall malaria incidence, severe morbidity and mortality, to eliminate malaria transmission in the urban area of state capitals across the Amazon, and to prevent resurgence in malaria-free areas. The Intensification Plan of Malaria Control Activities in the Legal Amazon (PIACM) targeted 254 municipalities (32.1 % of the total number of municipalities in the Amazon), which gathered 93.6 % of the malaria cases. The criteria for selection of those municipalities were: (i) those that had an annual parasite index (API) equal or greater than 50 cases per 1000 people; (ii) those where *P. falciparum* malaria was responsible for 20 % or more of the total number of cases; (iii) the capitals of the nine States that comprise the Amazon region; (iv) the set of municipalities that accounted for at least 80 % of all malaria cases in each State; and (v) those where urban malaria was observed [54]. Control measures were tailored to each specific epidemiological setting. A 39 % reduction in malaria incidence, compared to 1999, was achieved by the end of 2001.

In 2003, the Ministry of Health launched the National Malaria Prevention and Control Programme (NMPCP) with the goal to reduce incidence, mortality and severe malaria cases, to eliminate malaria from urban areas in the state capitals, and to prevent reintroduction of transmission in areas where it had been interrupted [55]. While the goals of the NMPCP were broad, following the decentralization of the health system during the 1990s each municipality could adopt different control strategies. For example, free-of-charge distribution of long-lasting insecticide-treated bed nets (LLINs) started in the state of Acre in late 2006 [56], and was later expanded to all high-risk municipalities in the Amazon between 2009 and 2011. In the early twenty first century, countrywide malaria cases, severe morbidity, and lethality have decreased dramatically. In 2000, 615,247 cases were confirmed, with 21,288 hospitalizations and 243 deaths; in 2013 there were 179,236 cases with 2365 hospitalizations and 41 deaths [16].

Currently, the Amazon Basin has nearly 27 million inhabitants (13.4 % of the total population of Brazil). In 2014, 143,910 microscopically confirmed malaria cases and only 41 malaria-related deaths were recorded; this is the lowest incidence in 35 years (Fig. 3). About 60 % of all infections are diagnosed within 48 h after the onset of symptoms, preventing severe morbidity, and 16 % of the cases were identified through active case detection. Laboratory diagnosis of malaria by microscopy or rapid diagnostic tests (RDTs, which are mostly used in remote areas), is freely provided, being required for free treatment. A network of nearly 3500 malaria outposts, 4900 microscopists and 7900 health agents facilitate access to diagnosis and treatment even in remote communities. First-line treatments, provided at no cost in government-run malaria outposts, are CQ-PQ for *P. vivax*, and an artemisinin combination therapy (ACT)—currently artemether–lumefantrine—plus a gametocidal dose of PQ for *P. falciparum*. Anti-malarial drugs are not available in the private sector.

Based on those achievements, in November 2015 Brazil was awarded by PAHO the Malaria Champions of the Americas Award. In the same month, the NMCP of the Ministry of Health launched the Plan for Elimination of Malaria in Brazil. The plan is part of the Sustainable Development Goals [57] launched by the United Nations, with the goal to reduce the global number of cases by 90 % until 2030, and to eventually eliminate malaria in 35 countries. The Brazilian plan focuses on *P. falciparum* malaria, and provides guidelines to municipalities on diagnostics, treatment, vector control, and community sensitization and education [58, 59]. Next, several potential challenges that may impact the elimination efforts in Brazil are discussed.

### Challenges for malaria elimination

#### Symptomless infections

The natural history of *P. falciparum* malaria has been well characterized in areas of high endemicity in Africa. Children have a primary malaria attack during their first year of life, while most toddlers and juveniles have already developed resistance against severe disease, but still experience a few clinical episodes. African adolescents and adults, in contrast, are often clinically immune; they remain free of malaria symptoms despite continuous exposure to the parasite, but carry parasites throughout the transmission season. Life-long exposure to malaria parasites rarely leads to sterile immunity; low-density blood-stage infections remain detectable with molecular techniques, such as polymerase chain reaction (PCR), in all age groups. Nevertheless, little is known regarding the acquisition of clinical immunity to malaria in areas of less intense malaria transmission in Latin America.

One of the first reports of asymptomatic malaria infections in Brazil came from surveys by Avery-Jones and Ferreira Neto [60] in the costal belt of Santa Catarina State (Southern Region), where *P. vivax* was the dominant species. In this typical “bromeliad-malaria” setting covered by Atlantic rainforest (see below), the only known malaria vectors were *Anopheles (Kerteszia) cruzi* and *Anopheles (K.) bellator*, which breed in water trapped in bromeliad plants [61]. Using conventional microscopy, the investigators found 39 individuals to be parasitaemic at one or more monthly house-to-house surveys, but only one of them had a malaria-related illness at the time of diagnosis. Asymptomatic parasite carriage persisted for up to 6 months. Moreover, nine symptomless relapses were diagnosed following CQ treatment of 13 symptomatic infections [60]. Similarly, low-density asymptomatic *P. vivax* infections, some of them initially missed by conventional microscopy but indirectly diagnosed by serology [62, 63], have also been reported in other bromeliad-malaria settings in southeast Brazil [16]. Subclinical infections were later described in Amazonians living in traditional riverine communities and exposed to malaria since birth [64–66]. These symptomless infections were found to be four to five times more prevalent than the symptomatic ones in riverine communities in Rondônia, with the risk for developing symptomatic malaria being inversely correlated with the subjects’ age [64].

In contrast, all malaria infections, even those with very low parasite densities, were thought to elicit clinical disease in malaria-naïve migrants settled on the Amazon frontier [67–70]. Molecular diagnosis, however, has often revealed entirely symptomless, low-density infections in migrant subjects living in typical frontier settlements in the Amazon [71–73]. For example, 56.6 % of all *P. vivax* infections diagnosed over 3 years in newly occupied farming settlements in Amazonas State were found to be asymptomatic; one-third of them were both submicroscopic (i.e., missed by microscopy) and asymptomatic [70]. Apparently healthy subjects accounted for half of the total *P. vivax* biomass found in the local population and nearly all asymptomatic *P. vivax* carriers had mature gametocytes detected with molecular techniques. Only 17.0 % of the asymptomatic *P. vivax* carriers developed clinical symptoms over 6 weeks of follow-up, becoming detectable by routine surveillance of febrile illnesses [73]. Similar findings were reported during the follow-up of asymptomatic parasite carriers in another Amazonian settlement [74]. After five to 8 years of continuous exposure to low/moderate levels of malaria transmission, the prevalence or incidence of both infection and malaria-related disease decreased steadily among native Amazonians and migrants, suggesting the acquisition of not only anti-disease immunity but also some degree of anti-parasite immunity [65, 73].

The infectiousness of gametocytaemia in asymptomatic carriers remains largely unknown. Avery-Jones and Ferreira-Neto [60] suggested that even a small but constantly present number of symptomless individuals could result in a large number of infective man-days. Specifically, if four infective asymptomatic gametocyte carriers were present during all 365 days of the year, a total of 1460 infective man-days would be observed. Similarly, if 10 asymptomatic gametocyte carriers were present during all 365 days of the year, a total of 3650 infective man-days would be observed. However, assuming that individuals would remain infective for 6 days, and considering all 170 laboratory-confirmed symptomatic malaria cases that had been diagnosed over a period of 12 months in their study site in Santa Catarina, only 1020 infective man-days would be observed. Nevertheless, the infectiousness of asymptomatic carriers of low-grade parasitaemia to Amazonian malaria vectors remains understudied; one study described an infection rate of 1.2 % after feeding *An. darlingi* with blood from asymptomatic *P. vivax* carriers, and of 22 % for the mosquitoes fed with blood from symptomatic carriers [75].

With regards to control activities, the challenge is how to detect asymptomatic infections in order to minimize the number of new infections originating from them. Mass blood surveys can detect asymptomatic malaria, but as transmission declines large populations would have to be screened to diagnose relatively few asymptomatic carriers, reducing the cost-effectiveness of this approach [60]. An alternative is to conduct reactive active case detection so that a confirmed malaria infection triggers the screening of individuals in a defined neighbourhood [76]. Regardless of the detection strategy, the diagnostic techniques available for large-scale use, such as microscopy and RDT, may not be sensitive enough to detect low-grade infections that are typical of residual malaria settings [77].

A reactive active case detection strategy with sensitive molecular diagnostic methods to detect new *P. vivax* infections in the neighbourhood of malaria cases diagnosed by routine surveillance (index cases) is currently being tested in Brazil. This strategy has been tailored for *P. vivax* infections, which tend to be maintained at low parasite densities and may relapse despite radical treatment. Four case-detection rounds (0, 30, 60 and 180 days after the index case diagnosis) were carried out within a 3 km radius around the index case to capture individuals during their primary infections and relapses. Molecular barcoding using single-nucleotide polymorphisms (SNPs) [78] or microsatellites [79] will facilitate the analysis of genetic relatedness between patient-derived *P. vivax* samples to elucidate malaria transmission networks.

#### Eliminating bromeliad malaria in southern Brazil

**Fig. 4:**
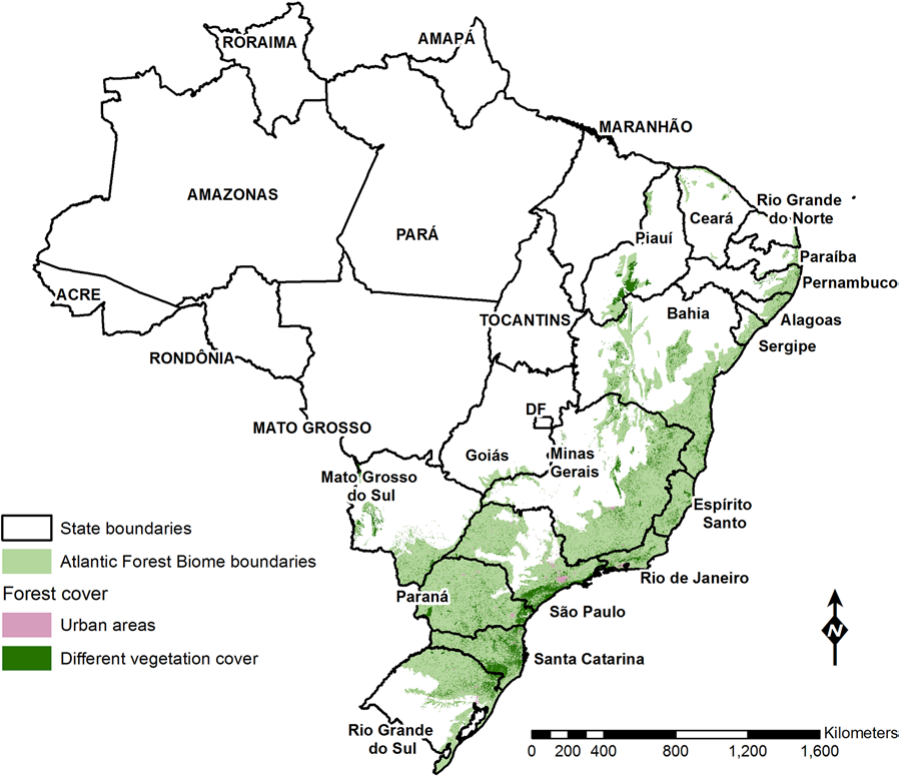
Area covered by the Atlantic Forest biome in Brazil, where pockets of bromeliad-malaria transmission persist. The extension of the Atlantic forest biome was defined by a federal law in 2006. More than 60 % of the population lives in large urban centers located in the Atlantic forest biome, thus, of the original biome area (which represents 17.4 % of the Brazilian territory), only about 8 % remains as forest. States that compose the Amazon region have their names written in* uppercase*

A small proportion (about 0.05 %) of all autochthonous malaria cases currently recorded in Brazil originate from areas along the Serra do Mar mountain range that extends across the states of Rio de Janeiro, Minas Gerais, Espírito Santo, São Paulo, Paraná, and Santa Catarina, in the Southeast and South regions, and is covered with by the Atlantic rainforest biome (Fig. 4) [16]. In these areas, the main (or only) malaria vectors are *An. cruzi* and *An. bellator*, which breed in water trapped by the leaf axils of bromeliad plants that are particularly abundant in the Atlantic rainforest [61]. Initially called “forest malaria” [80], and later described as “bromeliad malaria” [81, 82], malaria transmitted by *Kerteszia* anophelines was historically endemic in coastal areas of Southeast and South Brazil [83]. Bromeliad malaria is currently uncommon in most of the Atlantic forest biome of Brazil, but eliminating residual transmission in remaining foci, which involve non-human primates as parasite reservoirs, has been a major challenge for several decades.

Only *P. vivax* and *P. malariae* infections are currently reported in humans in bromeliad malaria settings in Brazil, where monkeys of the genus *Alouatta* (howler monkeys) and *Cebus* (capuchin monkeys) are naturally infected with these malaria parasites and may represent major animal reservoirs of parasites [84]. However, *P. falciparum* was nearly as prevalent as *P. vivax* in bromeliad malaria areas of Santa Catarina State in the late 1940s [60]. Furthermore, *P. falciparum* infection was recently reported in *An. cruzi* vectors from São Paulo State [85], suggesting that *Kerteszia* anophelines may also transmit *P. falciparum*.

Bromeliad malaria constituted a major public health challenge in Santa Catarina, with over 26,000 laboratory-confirmed infections diagnosed in 1947 in a population of 670,000 [60]. Local transmission of *P. falciparum* and *P. malariae* ceased in 1956 and 1961, respectively, as a result of control activities that included tree removal near major towns and replacement with eucalyptus trees (in which bromeliads do not grow) and vegetable/flowering plants, manual removal of bromeliad plants, intensive DDT spraying, and prompt diagnosis and CQ treatment of laboratory-confirmed infections [86, 87]. Nevertheless, *P. vivax* transmission persisted in the northern coast of Santa Catarina until the mid-1980s, where subpatent and asymptomatic human infections remained relatively common.

Prior to the molecular diagnosis era, serology was used to identify subjects with evidence of current or recent infections in bromeliad malaria foci along the coast of northern Santa Catarina [18]. In 1980–1982, over 20,000 subjects living in endemic sites in the towns of São Francisco do Sul and Araquari had finger-prick blood samples spotted onto filter papers and examined for malarial antibodies with the indirect fluorescent antibody test (IFAT), using *P. vivax*-infected red blood cells as capture antigens. The nearly 500 subjects with antibody titers ≥64, regardless of clinical symptoms, received a full course of CQ and PQ. No further autochthonous malaria cases have been reported in these two towns since 1986 [86]. Similarly, over 11,000 samples were tested for antibodies by IFAT in three consecutive population-based surveys carried out between 1984 and 1984 in rural sites surrounding the town of Peruíbe, southern São Paulo. Overall, 146 (1.3 %) samples tested positive (antibody titers ≥ 16). The 70 (0.6 %) subjects with antibody titers ≥64 were treated with CQ and PQ; 40 infections were further confirmed by thick-smear microscopy [63].

Interestingly, serology has the potential to detect not only ongoing blood-stage infections missed by conventional microscopy [62], but also recent infections, either symptomatic or not, that might have led to *P. vivax* hypnozoite formation in the liver. Despite this potential, the use of serology for targeting asymptomatic carriers of hypnozoites and blood-stage infections in areas approaching *P. vivax* malaria elimination has been rarely reported.

#### Submicroscopic infections

Average parasite densities tend to decrease, with higher proportions of *P. vivax* infections being missed by microscopy but detected by PCR as malaria prevalence decreases in Amazonian communities [73]. Accordingly, between half and three-fourths of all PCR-diagnosed infections are typically missed by microscopy in hypo- and mesoendemic settings in Brazil [64, 65, 72, 73]. One-fourth of all *P. vivax* infections diagnosed in one of these settings had densities <10 parasites per microlitre of blood [73], being likely to be missed even by experienced microscopists. Similarly, the proportion of *P. falciparum* infections that are submicroscopic but still detectable by nucleic acid amplification (NAA) increases in Africa, from 20 % in areas with the highest transmission to 70–80 % in areas with the lowest transmission [88].

From a clinical perspective, these chronic, low-density infections may not be entirely harmless. For example, subpatent parasitaemia has been associated with increased risk of anemia in riverine Amazonian populations [65]. From a public health perspective, the most important question is to what extent submicroscopic infections contribute to residual malaria transmission, arguing for the use of more sensitive diagnostic techniques in the context of malaria elimination [89]. In low-prevalence settings in Africa, carriers of a submicroscopic *P. falciparum* parasitaemia were estimated to be the source of 20–50 % of all mosquito infections [88]. However, no comparable estimates are currently available for *P. vivax* in the Americas. Accordingly, WHO acknowledges the need for “more research to understand better the contribution of submicroscopic infections in malaria transmission in low endemic settings and to identify which diagnostic strategies and NAA-based diagnostic techniques are most cost-effective in accelerating malaria elimination, compared to conventional malaria elimination methods” [89].

Serological tests that detect malaria-specific antibodies are currently unable to differentiate between recent and old infections. Therefore, they play no major role in routine case finding and management. However, in very low transmission settings serology helps to identify subjects who have been recently exposed to *P. vivax* and may still carry hypnozoites or even a low-grade (mostly asymptomatic) asexual blood-stage parasitaemia, that are missed by microscopy [62, 63]. The bromeliad-malaria elimination campaigns in southern Brazil, as discussed before, illustrate how serology can be useful for targeting human reservoirs of *P. vivax* in areas of residual transmission. Moreover, in communities where malaria has been eliminated, serology may help to identify resurgent malaria, especially when children and young adults, who were born after transmission was interrupted, are found to have specific antibodies.

#### Evolving *Plasmodium falciparum* anti-malarial drug resistance

In 1825, physician José Maria Bomtempo reported that quinine was used indiscriminately and at very high dosages in Rio de Janeiro [90]. Indeed, the first evidence of malaria parasite’s resistance to quinine came from Brazil in the early 1900s. Arthur Neiva, in 1907, diagnosed malaria infections in individuals under compulsory prophylaxis with quinine (500 mg every 2 or 3 days) who worked in highly malarious marshlands 60 km away from Rio de Janeiro, the country’s capital at that time [91]. He thus recommended daily 500 mg doses of quinine as the only effective chemoprophylaxis in that area [91]. Miguel Couto [92] reported the use of intravenous methylene blue to treat quinine-resistant malaria infections acquired in Rio de Janeiro as well as in the Amazon, but he did not describe the quinine dose initially used. The finding by Oswaldo Cruz of quinine-resistant malaria among workers in the Madeira-Mamoré railway construction site located in Rondônia state, western Amazon [93], was soon confirmed by Bernhard Nocht and Heinrich Werner, who documented quinine failures during the treatment of German workers returning from that region to Hamburg in 1910 [94]. Interestingly, despite these early reports, quinine remained widely used in Brazil until the mid-1990s, usually in association with tetracycline or doxycycline, to treat uncomplicated *P. falciparum* infections. Relatively little evidence of in vitro [95, 96] or in vivo resistance to quinine [97, 98] has been published since then.

Emerging resistance to CQ and a fixed-dose sulfadoxine–pyrimethamine (SP) combination may have greatly contributed to explosive *P. falciparum* malaria outbreaks across the Amazon in the 1980s. The first report of CQ failure to clear *P. falciparum* infections in Brazil, presented at a national medical conference in 1954, drew relatively little attention [99], but later publications documented CQ resistance in Brazil [100, 101] and Colombia [102] in the early 1960s. At least two CQ-resistant founder populations have been identified in this region, one circulating in the Amazon Basin of Brazil and Peru, and the second in Ecuador and Colombia [103]. CQ resistance became widespread across the Amazon Basin by the mid-1980s [104], when malaria cases were on the rise associated with colonization projects and environmental changes in the region (Fig. 3).

SP had been available in Brazil since the 1960s to treat CQ-resistant falciparum malaria [105]. SP-resistant *P. falciparum* strains emerged within a few years, being first documented in the Centre-West region in 1972 [106]. By the end of the 1980s, SP failed to cure >90 % of *P. falciparum* infections in the Amazon [99], and since 1990, SP is no longer recommended for malaria chemotherapy in Brazil [99]. Before the introduction of ACT in 2006 [107], the first-line regimens for uncomplicated falciparum malaria recommended by the Ministry of Health of Brazil were quinine plus doxycycline (formerly tetracycline) for 7 days or a single dose of mefloquine. Case reports of mefloquine (e.g., [108]) and quinine–tetracycline [e.g., 109] failure in falciparum malaria have been published since then. The few clinical trials that evaluated the therapeutic efficacy of these regimens reported cure rates ranging between 98.9 [110] and 100 % [111] for mefloquine, and between 77.3 [98] and 100 % [97] for quinine plus tetracycline.

ACT has gradually replaced quinine–doxycycline and mefloquine as the first-line regimen for uncomplicated falciparum malaria in Brazil in the late 2000s. Nearly 24,000 *P. falciparum* infections were treated with a fixed-dose artesunate–mefloquine (ASMQ) combination in Juruá Valley, Acre, between July 2006 and December 2008. Following this large-scale intervention, *P. falciparum* malaria incidence rates decreased substantially, with lower hospital admission rates, and a reduced proportion of *P. falciparum* to *P. vivax* infections in this region [112]. A recently completed trial showed no evidence, either clinical or molecular, for emerging *P. falciparum* resistance to the fixed-dose ASMQ combination in Juruá Valley [113]. More data on ACT efficacy in other areas of Brazil are expected to be made publicly available by the Amazon Network for the Surveillance of Antimalarial Drug Resistance (RAVREDA), a network organized in 2001 by the Amazonian countries of South America, with PAHO support, to respond to the challenge of anti-malarial drug resistance in the region. RAVREDA operates in close association with the Amazonian Malaria Initiative (AMI), a partnership of the United States Agency for International Development with PAHO, the Centers for Disease Control and Prevention (CDC), the Management Sciences for Health’s Rational Pharmaceutical Management Plus (MSH/RPM Plus) programme, the United States Pharmacopoeia, and Links Media, Inc.

Resistance to artemisinin derivatives has not been documented in Brazil, but there is some concern that resistant parasites may circulate in areas of Suriname, Guyana and French Guyana bordering Brazil. Suriname has recently attracted a large number of immigrants, mainly from Brazil, to work in gold mines in the eastern and central parts of the country. An estimated 15,000 (mostly illegal) miners currently live in settlements where health infrastructure and compliance with national malaria treatment policies are typically very poor [114]. A recent study in Suriname has documented a large proportion (31 %) of *P. falciparum*-infected patients treated with artemether–lumefantrine who remained parasitaemic by day 3, consistent with some degree of artemether resistance in Suriname [115], although these findings have not been confirmed in other areas of the country. Nevertheless, Suriname is complying with WHO recommendations for malaria control and *P. falciparum* malaria is now rarely diagnosed in the country [116]. In Guyana, the *kelch propeller domain* (*k13*) gene mutation C580Y, associated with artemisinin resistance, has been found in 5 % of 98 *P. falciparum* isolates analysed [117]. Interestingly, an analysis of polymorphisms upstream and downstream the *k13* gene suggest that the C580Y mutation has emerged independently in Guyana and Southeast Asia [117]; these mutant parasites, however, did not have their drug sensitivity patterns analyzed so far. In French Guyana, up to 15,000 illegal gold prospectors live in high-endemic areas along with 30,000 legal residents. They typically use erratic ACT-based regimens, potentially including fake or substandard drugs, to treat *P. falciparum* infections, favouring the selection of drug-resistant strains. Artemisinin resistance had not been characterized so far in French Guyana, but the country fails to comply with WHO recommendations for anti-malarial treatment [116]. Therefore, unequal cross-border efforts to control malaria and contain the emergence of anti-malarial drug resistance pose potential threats to eliminate the disease from French Guyana, Brazil and Suriname [116].

#### *Plasmodium vivax* resistance to chloroquine

The global spread of CQ-resistant *P. vivax* strains [118] can further complicate malaria elimination efforts in Brazil. Radical cure of *P. vivax* malaria is achieved with 25 mg/kg of CQ base over 3 days (maximum adult dose, 1.5 g over 3 days), combined with a short hypnozoitocidal regimen of 0.5 mg/kg/day of PQ base (maximum daily dose, 30 mg/day) over 7 days in patients that weight under 70 kg. Since subtherapeutic PQ doses may lead to relapses in overweight patients [119], weight-adjusted PQ doses are now recommended in Brazil for patients over 70 kg [120].

CQ-resistant vivax malaria has been reported in Colombia and Brazil since in the late 1980s, but anti-malarial treatment had not been supervised, and drug levels were not measured, in these early studies [reviewed by 121]. The first solid evidence of CQ-resistant *P. vivax* infections in South America was published in 1996. Two Canadian patients returning from Guyana had received CQ and PQ, with therapeutic CQ levels documented at the time of parasite recurrence [122]. Further evidence of *P. vivax* resistance, confirmed by CQ level measurements, originated from patients from Peru given CQ alone in 2003 [123]. Two studies described *P. vivax* resistance to CQ in Manaus, the major port city in the Amazon Basin of Brazil, in the 2000s. The first study documented *P. vivax* recrudescence, despite adequate plasma levels of CQ, in 11 of 109 (10.1 %) patients treated with CQ alone who were followed up for 28 days [124]. The second study reported *P. vivax* recurrences in seven of 135 (5.2 %) patients treated with CQ and PQ who were followed up for 28 days; all of them had CQ levels above 100 ng/mL of whole blood at the time of recurrence [125]. It remains to be determined whether CQ resistance has spread to other areas in Brazil; a very recent clinical trial has detected little *P. vivax* resistance to CQ alone (2 %), and none to CQ–PQ combination, in major malaria hotspots in Juruá Valley (MUF, unpublished observations). Although ACT has been used to treat CQ-resistant *P. vivax* infections worldwide [126], its efficacy as an alternative to CQ remains unknown in Brazil.

The finding that PQ reverses CQ resistance in *P. falciparum* suggests that a similar effect might occur in *P. vivax* isolates simultaneously exposed to both drugs [reviewed by 127]. In fact, recent meta-analyses showed a lower efficacy of CQ alone against *P. vivax* asexual blood stages compared to CQ co-administered with PQ [118, 128]. Over the past 60 years, CQ and PQ have been routinely combined for the radical cure of *P. vivax* infections in most of South America, but not in Melanesia and Southeast Asia, where highly prevalent severe G6PD deficiency may lead to PQ-induced haemolysis. Indeed, CQ-resistant *P. vivax* emerged in regions where PQ is not widely used and remains less common in countries where CQ and PQ are routinely coadministered [118, 121], indicating that CQ–PQ combination therapy may have delayed the emergence of CQ-resistant *P. vivax* strains in selected settings.

PQ safety is an open question, since there is no practical, field-deployable rapid diagnosis test for G6PD deficiency screening prior to PQ administration [129]. Severe G6PD deficiency is uncommon across the Amazon Basin [130, 131], but severe haemolysis has been occasionally reported following PQ treatment [132]. Moreover, PQ cannot be administered to pregnant and breastfeeding women and to children less than 6 months of age, because the risk of haemolysis. PQ efficacy also remains largely undetermined in Brazil. Relapse rates between 14.0 and 24.5 % have been described following supervised CQ–PQ treatment of imported *P. vivax* infections in non-endemic sites [119, 133]. Furthermore, an imported *P. vivax* infection acquired in Brazil was found to relapse despite the administration of 900 mg of PQ over 30 days [134]. The extent to which PQ metabolism in Amazonian populations is affected by polymorphisms in cytochrome P450 (CYP) family members, such as CYP 2D6, remains unknown. In fact, reported failures of PQ may be partially associated with poor PQ metabolism in patients, rather than true drug resistance. However, the local monkey-adapted *P. vivax* strain Brazil I is PQ-resistant in monkeys [135]. If PQ is failing in Brazil, for whatever reason, a substantial proportion of *P. vivax* infections may actually be relapses, placing improved anti-relapse treatment as a top research priority for guiding malaria elimination in this country.

The finding that high-dose PQ regimens are safe and effective among G6PD-normal subjects led the US Centers for Disease Control and Prevention (CDC) to recommend 420 mg of PQ over 14 days as the standard anti-relapse regimen for adults in areas where standard PQ treatment fails [136]. WHO recommends a PQ dose of 0.5 mg/kg of body weight over 14 days to prevent relapses of infections acquired in Southeast Asia and Oceania [137], but this high-dose PQ regimen has not been evaluated in Brazil. Tafenoquine (TQ) is an alternative to PQ that requires shorter treatment regimens with improved compliance; a single dose of 300 mg TQ coadministered with CQ is effective to prevent *P. vivax* relapses in Brazil, Peru, India, and Thailand [138]. However, treatment with this long-lasting hypnozoitocidal drug does require prior screening for G6PD deficiency, an additional challenge due to the lack of a rapid screening test. Moreover, TQ is neither licensed nor available commercially in Brazil.

#### The hidden burden of malaria in pregnancy

Malaria infection during pregnancy is associated with substantial risks for the mother, her fetus, and the neonate. Stillbirth, intrauterine growth retardation, prematurity, low birth weight, spontaneous abortion, increased neonatal and maternal mortality, and reduced neurocognitive function later in childhood are documented complications of malaria in pregnancy (MiP) [139, 140].

Between 6000 and 9000 microscopy-confirmed MiP cases are recorded in Brazil each year [141]. This represents 4–6 % of all malaria cases in the country. However, since conventional microscopy and RDTs fail to detect a substantial proportion of MiP episodes that are later diagnosed by PCR [142], these figures are likely to be underestimated. Health professionals involved in antenatal care, such as nurses and obstetricians, usually do not perceive MiP as a major preventable and treatable cause of morbidity in pregnant women and their offspring. This is partially due to the fact that infections with *P. vivax* are associated with less severe clinical consequences in pregnant women than those with *P. falciparum*, although an increased risk of low birth weight and anemia was documented in a large cohort study in Southeast Asia [143]. Erythrocytes parasitized with the former species do not sequester massively in placental capillaries [144, 145], but some placental changes associated with MiP, such as syncytial knotting and increased thickness of the placental barrier, have been recently documented in Brazil and may affect fetal growth [145, 146]. Because PQ cannot be administered during pregnancy, relapses are common. Overall compliance with a weekly CQ regimen for suppressing relapses following *P. vivax* infection during pregnancy is usually poor in endemic areas of Brazil.

Almost one-third of the MiP cases recorded in Brazil between 2003 and 2012 were due to *P. falciparum,* although this species accounted for only one-fourth of the malaria burden in non-pregnant women during the same period [141]. Indeed, a cross-sectional survey of 1699 febrile women at childbearing age found a significantly greater *P. falciparum* to *P. vivax* ratio in pregnant women (1:2.3) than in their non-pregnant counterparts (1:5.6) living in Manaus, Brazil [147]. Also, a cohort study in the Peruvian Amazon found pregnant women to be twice more likely to have *P. falciparum* infections than non-pregnant women living in the same community, but no pregnancy-associated difference was found in the risk of *P. vivax* infections [148].

Pregnant women living in malaria-endemic areas of Brazil must be tested for malaria parasites at every antenatal care visit and receive supervised anti-malarial treatment whenever MiP is confirmed [141]. In areas with stable transmission of *P. falciparum* in Africa, pregnant women are encouraged to sleep under insecticide treated bed nets (ITNs), and at least two doses of intermittent preventive treatment (IPTp) with SP should be administered during antenatal care visits to prevent MiP [149]. Although sub-Saharan Africa has high rates of antenatal care attendance, current IPTp coverage is still low [150], despite the fact that IPTp-SP is highly cost-effective for prevention of MiP and reduction of neonatal mortality, even in areas with some molecular evidence of SP resistance in *P. falciparum* populations [151]. Currently, there is no evidence to support the use of IPTp-SP to prevent MiP in areas with substantially lower levels of malaria transmission, such as those in Brazil.

#### Vector behaviour and control

Research conducted in the Amazon in 1931 showed that *An. darlingi* was found in much greater numbers indoors (endophilic) than outdoors (exophilic). Data collected between July 1942 and June 1946 in Rondônia (then called Guaporé territory) indicated that 93 % of the mosquitoes captured were present inside the houses. For the whole Amazon, this percentage was 88 % [152]. In 1977, another study showed that *An. darlingi* would enter houses that had been sprayed with DDT, but only to feed, and not to rest, as they would do in unsprayed houses [153]. Early records also show that *An. darlingi* was to some extent exophilic before the use of DDT [154]. Studies in Rondônia during the 1980s, however, found *An. darlingi* mainly outdoors, in the vicinity of houses [69, 153, 155, 156]. In addition to differences in biting location, *An. darlingi* also shows different patterns of peak biting time. For example, bimodal biting curves have been reported, with peaks in the early evening and at dawn, hours at which workers could be carrying out outdoor activities [156–158].

The exophilic behaviour is a point of discussion. Some argue that DDT use could have contributed to this transformation, selecting the exophilic strains to be more prevalent. Others consider that extensive deforestation and disorganized occupation could be indirectly responsible for modifications in the mosquito behaviour, subtracting its former sources of food (wild animals, who were scared away by the new settlers) and bringing man closer to its breeding places [3]. Population and mutation differences have been documented in South America (where *An. darlingi* is the dominant vector), with a marked north–south divide [159]. Although chromosomal and isoenzymatic studies show a high degree of heterogeneity, no morphological characteristics for separation of sub-species have been found [156].

This diversity in *An. darlingi* behaviour has important implications for vector control. First, an exophilic behaviour and peak biting times limit the impact that using ITNs could have in malaria transmission. Despite the evidence on ITN effectiveness [160], no large scale trial of bed nets have been conducted in the Amazon. However, there is evidence that targeting ITNs to specific areas based on vector behaviour can be an important strategy to add to a package of interventions to reduce malaria transmission. Two studies conducted in specific areas in Venezuela and Colombia showed efficacy in ITNs to reduce malaria risk [161, 162]. In Brazil, LLINs impregnated with permethrin were distributed across Juruá Valley (see Fig. 4) starting in late-2006 [163]. Although malaria incidence fell by 32 % over the next months, the intervention did not follow a systematic design to allow a proper impact evaluation, controlling for other ongoing interventions. Also, two additional rounds of LLIN distribution (2010, 2014) led to little changes in malaria rates. Second, with regard to IRS, limiting factors include *An. darlingi* behaviour and the quality of houses, particularly in rural areas—these are often made with poor construction materials, with partial walls and no windows [56]. Although IRS was largely used in Brazil starting in the mid-1940s, during the first elimination campaign (Fig. 1), currently it is restricted to areas that match certain technical and operational requirements defined by the Ministry of Health [164]. Third, the use of larvicides is restricted to specific water habitats that can be easily identified and reached, such as fish ponds [165].

In summary, vector ecology and behaviour coupled with the local environmental conditions in the Amazon bring about major barriers for the adoption of large-scale vector control strategies. Indeed, in 2014, about 3 % of the population in the Amazon was covered by treated nets, and 1 % covered by IRS [166]. Yet, targeted interventions should be considered as important additions to a range of control strategies to reduce malaria transmission, as long as decisions are based on local knowledge of vector behaviour.

#### Hitting foci of *Plasmodium falciparum* infection

**Fig. 5:**
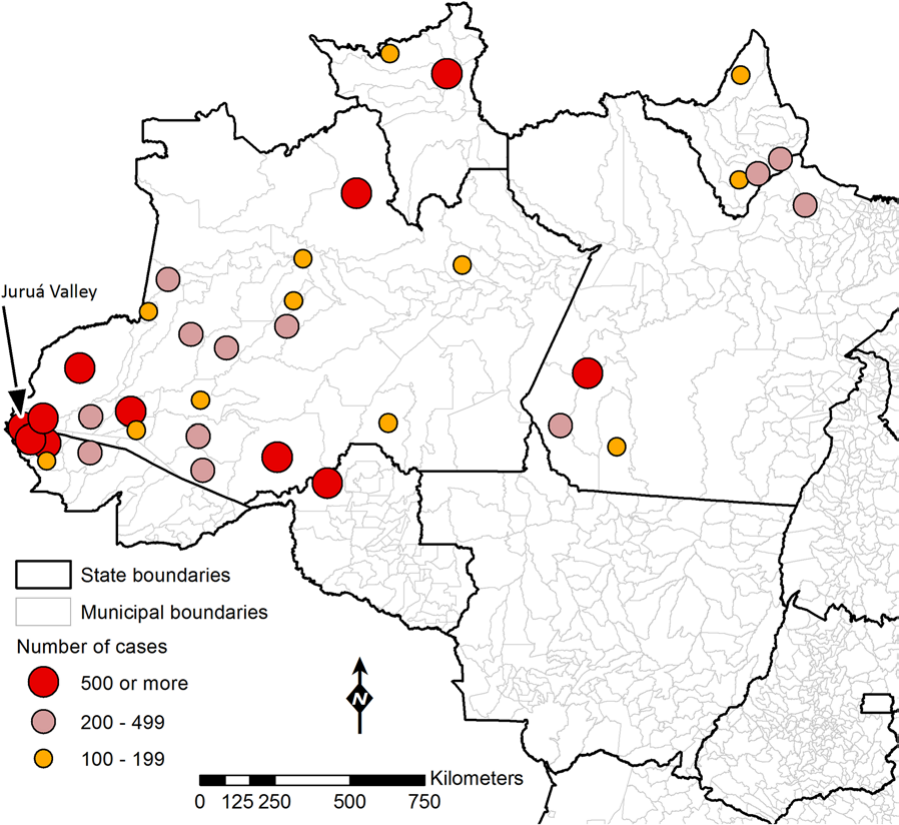
Current *Plasmodium falciparum* foci in Brazil. Municipalities indicated with *circles* in the map accounted for about 75 % of the laboratory-confirmed *P. falciparum* infections recorded in the Amazon in 2014. The* circle sizes* are proportional to the absolute number of cases in each municipality. Four high-risk municipalities (Cruzeiro do Sul, Mâncio Lima, Rodrigues Alves, and Guajará) are situated in Juruá Valley, westernmost Brazil, close to the border with Peru

Approximately 20,000 *P. falciparum* malaria cases were recorded in the Amazon Basin of Brazil in 2014. They were mostly restricted to ten high-risk areas, which account for three-fourths of the cases recorded countrywide (Fig. 5). Four municipalities in Juruá Valley (in the Western portion of the Amazon, close to the border with Peru), with a combined population of nearly 120,000 inhabitants, reported 46 % of all *P. falciparum* infections diagnosed in Brazil in 2014. The highly focal nature of *P. falciparum* malaria indicates that eliminating transmission of this species may be technically feasible by targeting a limited number of transmission pockets. Moreover, the potential of falciparum malaria for high morbidity and mortality and multidrug resistance, as well as its comparatively greater susceptibility to existing control measures, provide compelling arguments for prioritizing this species in malaria elimination strategies in Brazil. Indeed, the recently launched Plan for Elimination of Malaria in Brazil devotes special attention to *P. falciparum* malaria, and has the ultimate goal of eliminating the disease by 2030 [167].

As argued for the Greater Mekong Subregion in Asia [168], the window of opportunity for *P. falciparum* elimination may be rather short. First, an elimination strategy must be implemented before widespread anti-malarial drug resistance (more specifically, resistance to artemisinin derivatives) makes *P. falciparum* infections in Brazil untreatable with currently available drugs. In other words, “multidrug resistance is both an impediment to elimination and a reason for pursuing it” [168]. Second, financing and political commitment to malaria elimination tends to wave once *P. falciparum* becomes widely perceived as a relatively minor public health problem and other competing priorities emerge countrywide.

Eliminating residual *P. falciparum* transmission will require a comprehensive assessment of the factors that favor malaria transmission in well-defined foci across the Amazon. Improved local knowledge regarding the relative contribution of vectors (e.g., species distribution, abundance, behaviour, and insecticide resistance), parasites (e.g., drug resistance and virulence), humans (e.g., behaviour, health infrastructure, and patterns of mobility), and the environment (e.g., land use and land change) to persisting *P. falciparum* transmission will be crucial, especially since underlying drivers of transmission are not likely to be same across foci. Indeed, human-made vector breeding sites (e.g., fish pounds opened for commercial aquaculture) are a major factor leading to the current increase in *P. falciparum* malaria incidence in urban and peri-urban areas of Juruá Valley (Acre State, Western Amazon). Mining activities favor transmission in the outskirts of Itaituba (Pará State, Eastern Amazon), while in Barcelos (northern Amazonas State, Western Amazon) the majority of malaria cases is recorded in remote protected areas (mostly indigenous reserves). Finally, the main endemic sites in Lábrea (southern Amazonas State) are newly opened farming settlements. Thus, different strategies will be needed for controlling and eventually eliminating *P. falciparum* in each area.

At present, there is no evidence that artemisinin-resistant *P. falciparum* strains have been imported into Brazil. No data are available on anti-malarial drug resistance patterns of *P. falciparum* infecting Brazilians returning from mining camps in the Guiana Shield (a region comprising Guyana, French Guiana, Suriname and parts of Colombia, Venezuela and the northernmost tip of Brazil), where artemisinin resistance is suspected to be emerging [115].

#### Environmental changes

The natural environmental characteristics of the Amazon Basin offer suitable conditions for malaria transmission: temperature, humidity and the local vegetation guarantee a large population of vectors year-round, with less seasonal variation when compared to other areas [26]. Also, the water level of rivers increases dramatically during the rainy season, flooding areas immediately proximal to the margins, and as the water level decreases with the ending of the rainy season, pools of water suitable for mosquito breeding proliferate [169].

The massive deforestation observed in the Brazilian Amazon in the 1970s and 1980s brought about significant environmental changes and was associated with increases in malaria transmission, as previously discussed. In addition, large-scale development projects (e.g., road and dam construction) often produce environmental disturbances and social conditions that can be conducive to increases in vector-borne diseases, including malaria [170]. While expanding areas protected under environmental conservation policies would be beneficial to prevent an increase in malaria transmission [171], current trends make it reasonable to expect that deforestation in the Amazon will continue to be a reason for concern [172] driven by varied economic pressures (e.g., cattle ranching, agriculture, mining, and large-scale development projects). To mitigate some of those problems, since 2001 the Brazilian legislation requires that, as part of the licensing of development projects located in malaria endemic areas, an evaluation should be conducted by the Ministry of Health [173].

Considered as the biggest global health threat of the twenty first century [174–176], climate change is likely to have an impact on malaria, despite contrasting results of global models [177–182]. Climatic effects on malaria can occur directly, through extreme events (e.g., drought, flooding), increases in average temperature, and changes in precipitation patterns, but also indirectly, by population displacement, and water and food insecurity, which impact individuals’ exposure and vulnerability to infections. Despite the uncertainty embedded in climate change scenarios [183], it is expected that impacts on malaria will be observed, although the impacts and the responses are unlikely to be uniform across and within regions [184]. The magnitude of the impact will depend on: (i) temperature variability [185]; (ii) countries’ capacity to anticipate future changes; (iii) adaptation strategies adopted at varied scales; (iv) ongoing malaria control interventions; (v) the possibility that local characteristics that affect malaria risk can be augmented by climatic changes; and (vi) the level of clinical immunity among the population [178]. Since 1996, more than a dozen atypical years (El Niño/La Niña), and at least one extreme drought not at all related to El Niño/La Niña, were observed in the Amazon. Yet, their potential effect on the patterns and level of malaria transmission remains largely unknown.

#### Deploying effective surveillance

Surveillance can be defined in different ways [186]. Considering it as an intervention through which data are systematically collected, analysed, and interpreted, so that both symptomatic and asymptomatic cases are identified, triggering quick action to prevent further transmission [187], then the main challenge is how to properly identify all the cases. Among the potential problems in achieving proper surveillance are human mobility, border issues, asymptomatic infections, and weak health and information systems.

With respect to information systems, Brazil is in a privileged position. Since malaria is one of the diseases that require compulsory notification in the country, a national system gathers information on all malaria tests performed across the country (microscopy and RDT), with demographic information on patients, and with detailed test results. Data are entered locally, and available real-time at the municipal, state and federal levels.

Migratory movements have important malaria implications depending on the levels of immunity among migrants, the underlying patterns of transmission in sending and receiving areas, the local characteristics observed in areas of origin and destination, the individual knowledge about the disease, and the pattern of mobility [188, 189]. Human mobility is intense in the Brazilian Amazon driven by new economic opportunities (e.g., mining, land availability) or by the need to obtain goods and/or services [190]. In a context of intense human mobility, the challenge of asymptomatic infections is likely to be augmented, since symptomless migrants represent a mobile and silent reservoir of malaria infections. Moreover, human movements can facilitate the reintroduction of malaria in areas that have significantly reduced (or even eliminated) malaria. Critically to that is when unequal control efforts are adopted by neighbouring countries, potentially resulting in a high prevalence of imported cases near the borders [191, 192]. In that regard, one of the critical challenges in the Amazon region is the border between French Guiana, Suriname, and Brazil, where intense mobility (and importation of malaria cases) is fueled by gold mining in French Guiana and Suriname [116, 193].

## Conclusion

The Plan for Elimination of Malaria in Brazil, launched in November 2015, is in alignment with the new Sustainable Development Agenda [194], and builds on a unique momentum: the number of malaria cases in the country has reached its lowest levels in 35 years, *P. falciparum* is highly focal, and the geographic boundary of transmission has considerably shrunk. While the prospects for success are good, challenges are many and varied. This paper discussed the challenges ahead, some already present, others a possibility.

In light of the potential challenges, a few issues require some reflection. First, the promotion of collaboration between different government and private sectors (such as, for example, education, agriculture, finance, urban planning, transportation, and environment), albeit often difficult to achieve, is of paramount importance to add a development dimension to malaria control [195]. Brazil has already promoted important initiatives in the context of agrarian reform and infrastructure projects in areas suitable to malaria [196], but there is a need and an opportunity for additional collaborations.

Second, as malaria transmission continues to decline, guaranteeing resources for malaria interventions may clash with other pressing and emerging health priorities in the country. In that regard, history makes it very clear that malaria resurgence is a real possibility [197], and it should be avoided. Therefore, sustaining achieved gains, and moving towards further declines is the only option, and multisectoral collaboration can be an important ally in this task.

Third, individual risk perception of malaria and behaviour issues may affect the uptake of interventions and hamper the goals of elimination. Sensitizing individual and communities to maintain or adopt new behaviours that reduce the risk of infections is critically important as areas reach pre-elimination stages. New ways to promote behaviour change campaigns under scenarios of low transmission is an area that needs special attention [198].

Fourth, there is an increasing need for control measures that are tailored for *P. vivax,* the “last parasite standing”. Better strategies for relapse prevention in the presence of G6PD deficiency, and improved point-of-care laboratory diagnosis of low-level *P. vivax* parasitaemias are required. Helping to fill these knowledge gaps is an important contribution that Brazilian researchers can provide to the current nationwide malaria elimination efforts.

The next 5 years will be critical for elimination goals in the Americas region. Successful outcomes of the malaria elimination plan that Brazil just launched will inspire and provide evidence for similar efforts in other countries. Eventual setbacks in the current trends in malaria transmission in Brazil may suggest the need to fine tune the proposed plan, and may shed light on unforeseen challenges to achieve elimination. How this chapter in the history of malaria control in Brazil will end is highly awaited by the malaria community.

## Abbreviations

ACT: artemisinin combination therapy
AMI: Amazonian Malaria Initiative
API: annual parasite index
ASMQ: artesunate–mefloquine
CDC: US Centers for Disease Control and Prevention (CDC)
CQ: chloroquine
DDT: dichloro-diphenyl-trichloroethane
G6PD: glucose-6-phosphate dehydrogenase
IFAT: indirect fluorescent antibody test
INCRA: National Institute for Land Reform of Brazil
IPTp: intermittent preventive treatment
IRS: indoor residual spraying
ITN: insecticide-treated net
k13: kelch propeller domain
LLIN: long-lasting insecticide-treated bed net
MiP: malaria in pregnancy
NAA: nucleic acid amplification
NMPCP: National Malaria Prevention and Control Programme
PAHO: Pan American Health Organization
PCMAM: Amazon Basin Malaria Control Programme
PCR: polymerase chain reaction
PIACM: Intensification Plan of Malaria Control Activities in the Legal Amazon
POLONOROESTE: Northwest Region Integrated Development Programme
PQ: primaquine
RAVREDA: Amazon Network for the Surveillance of Antimalarial Drug Resistance
RDT: rapid diagnostic test
SNP: single-nucleotide polymorphisms
SP: sulfadoxine–pyrimethamine
USAID: US Agency for International Development
TQ: tafenoquine
WHO: World Health Organization
